# Structure and stability of different triplets involving artificial nucleobases: clues for the formation of semisynthetic triple helical DNA

**DOI:** 10.1038/s41598-023-46572-4

**Published:** 2023-11-07

**Authors:** N. R. Jena, P. K. Shukla

**Affiliations:** 1 Discipline of Natural Sciences, Indian Institute of Information Technology, Design, and Manufacturing, Dumna Airport Road, Khamaria, Jabalpur 482005 India; 2 Department of Physics, Assam University, Silchar, Assam 788 011 India

**Keywords:** Biophysics, Chemical biology, Computational biology and bioinformatics, Molecular biology, Structural biology, Health care, Chemistry

## Abstract

A triple helical DNA can control gene expression, help in homologous recombination, induce mutations to facilitate DNA repair mechanisms, suppress oncogene formations, etc. However, the structure and function of semisynthetic triple helical DNA are not known. To understand this, various triplets formed between eight artificial nucleobases (P, Z, J, V, B, S, X, and K) and four natural DNA bases (G, C, A, and T) are studied herein by employing a reliable density functional theoretic (DFT) method. Initially, the triple helix-forming artificial nucleobases interacted with the duplex DNA containing GC and AT base pairs, and subsequently, triple helix-forming natural bases (G and C) interacted with artificial duplex DNA containing PZ, JV, BS, and XK base pairs. Among the different triplets formed in the first category, the C-JV triplet is found to be the most stable with a binding energy of about − 31 kcal/mol. Similarly, among the second category of triplets, the Z-GC and V-GC triplets are the most stable. Interestingly, Z-GC and V-GC are found to be isoenergetic with a binding energy of about − 30 kcal/mol. The C-JV, and Z-GC or V-GC triplets are about 12–14 kcal/mol more stable than the JV and GC base pairs respectively. Microsolvation of these triplets in 5 explicit water molecules further enhanced their stability by 16–21 kcal/mol. These results along with the consecutive stacking of the C-JV triplet (C-JV/C-JV) data indicate that the synthetic nucleobases can form stable semisynthetic triple helical DNA. However, consideration of a full-length DNA containing one or more semisynthetic bases or base pairs is necessary to understand the formation of semisynthetic DNA in living cells.

## Introduction

Although DNA is traditionally known to store genetic information^1,2^ and control cellular functions^1,2^, it can be engineered for various technological applications (DNA technology), such as nanotechnology^3^, biosensing^4^, drug delivery^5^, forensic purpose^6^, agriculture^7^, gene silencing^8^, information technology^9^, medicine^10^, and vaccines^11^. For these technological applications, either DNA sequences would be modified, or new synthetic nucleotides would be inserted into the duplex DNA^3–11^.

Although most DNA technological applications involve single-stranded or double-stranded DNA, triple helical DNAs can be realized in the laboratory for different technological applications^12^. The formation of triple helical DNAs inside living systems is also possible by bending one of the strands of the duplex DNA at a mirror repeat sequence containing purine-rich (*H-DNA) or pyrimidine-rich (H-DNA) sequences^13^. Such triple helical DNAs are referred to as intramolecular triplexes, which can induce transcriptional repression and site-specific mutagenesis^13^. Triple helical DNA can also be formed due to the binding of a triplex-forming oligonucleotide (TFO) with the duplex DNA in sequence-specific manure. These triple helical DNAs are referred to as intermolecular triplexes and are highly stable (half-lives are of the order of days)^13^. In the case of the intermolecular triplexes, the TFOs contain natural (G, C, A, and T) or modified (5-methyl-cytosine) oligonucleotides and can be used to deliver drugs, silence disease-making genes, induce DNA repair pathways, promote site-specific mutations, and affect DNA replication^13–17^.

**Figure 1:**
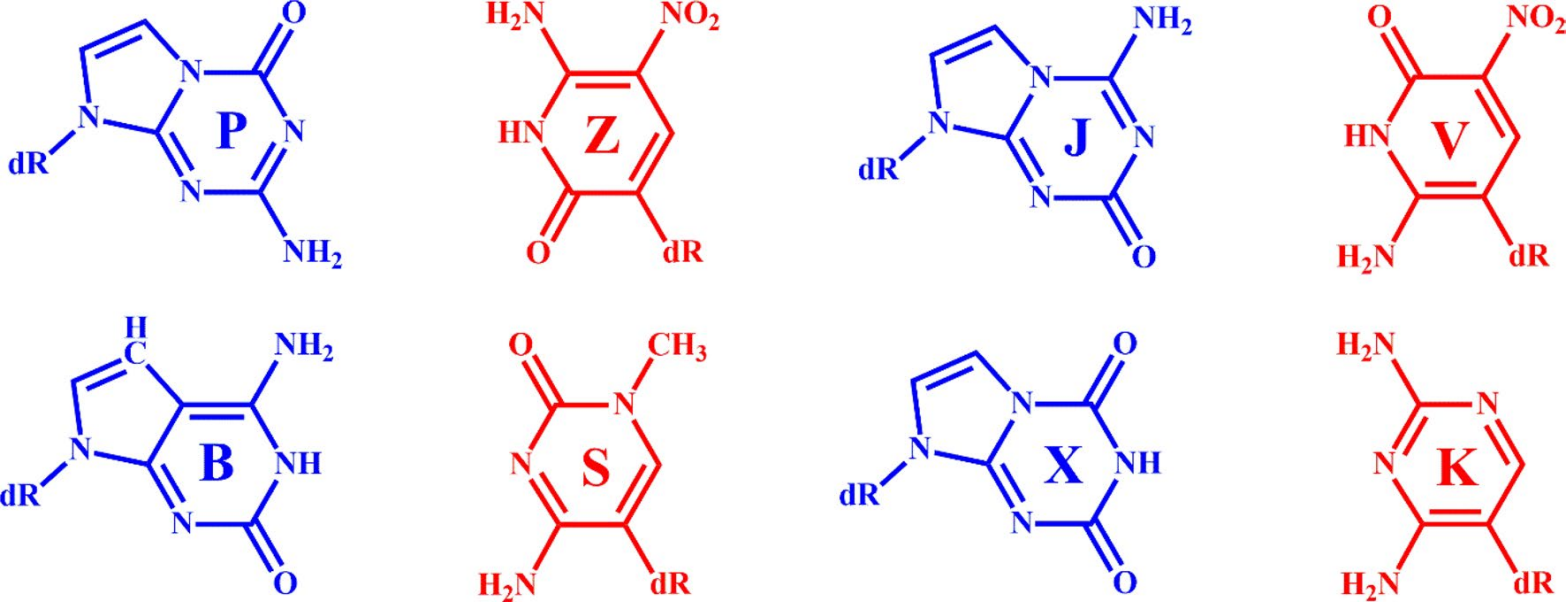
Structures of the eight artificial nucleotides. The N- and C-deoxy glycosidic bonds are represented by dR, which was replaced by an H atom during the geometry optimizations. The artificial purines (P, J, B, and X) are shown in blue, and the pyrimidines (Z, V, S, and K) are shown in red.

Recently, eight second-generation artificial nucleotides, such as P, Z, J, V, B, S, X, and K (Fig. 1) were proposed to expand the genetic information system^18–30^. These artificial nucleotides were proposed to produce stable DNA by making complementary non-canonical Watson–Crick base pairs like the natural GC pair. For example, the base pair interactions between P and Z, J and V, B, and S, and X and K were found to be about 1–3 kcal/mol more stable than the GC pair^25^. The base pair interactions between different analogs of P and Z were also found to be about 3–16 kcal/mol more stable than the GC pair^26^. Further, recently, a 16-mer DNA duplex containing six consecutive PZ pairs was shown to produce a stable duplex DNA^22,23^. Moreover, these artificial nucleotides were proposed to act as antiviral agents and bind strongly to antiviral proteins to inhibit the replication of the viral genome^31^. They can also be used as DNA aptamers^29,30^ to target the toxic form of anthrax protective antigen^32^. However, their usefulness in producing triple helical DNA has not been explored yet.

As these nucleotides are proposed to be recognized and replicated by polymerases^33–35^, triple helical DNA containing these artificial nucleotides may function naturally inside living cells. Therefore, it is likely that TFOs containing natural oligonucleotides (G, C, A, and T) can bind to the semisynthetic DNA to form an intermolecular triple-helical DNA. It is also likely that TFOs containing artificial oligonucleotides can bind to a natural duplex DNA to form a stable intermolecular triple helical DNA. Hence it is necessary to understand the structural and energetic aspects of different triplets formed by the binding of natural nucleobases with the artificial base pairs and the binding of artificial nucleobases with the natural base pairs in detail. Further, unlike natural nucleobases that contain N-glycosidic bonds, some of the artificial nucleobases (X, Z, V, S, and K) contain C-glycosidic bonds (Fig. 1), and therefore, their binding patterns are expected to be different from the natural nucleobases in the duplex DNA.

**Figure 2:**
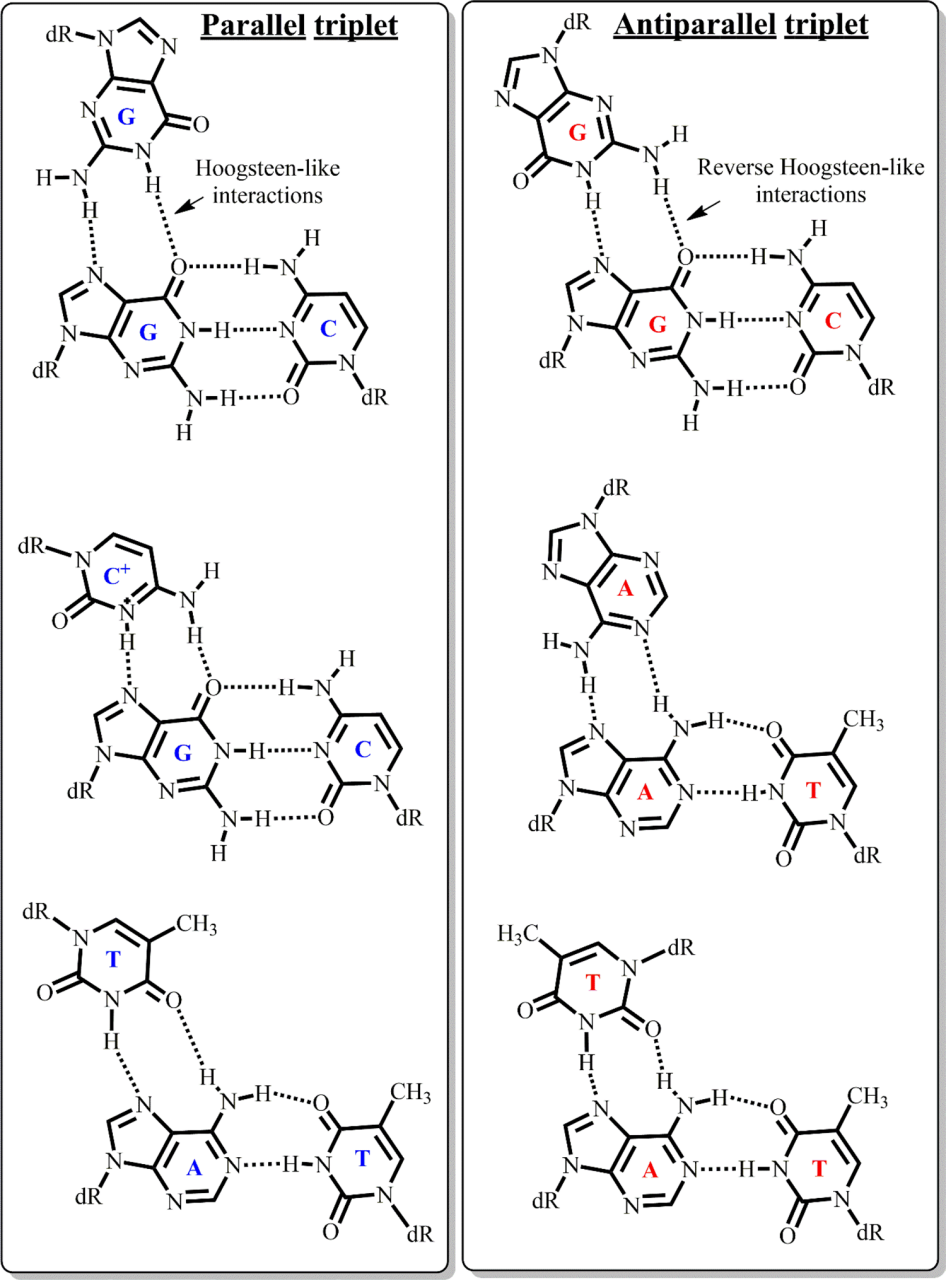
The structures of parallel and antiparallel triplets formed by Hoogsteen and reverse Hoogsteen interactions respectively between triplex-forming nucleotide and duplex-forming base pairs. Here dR represents the deoxy glycosidic bond, which was replaced by an H atom during the geometry optimizations.

Earlier, it was proposed that TFOs containing purines and pyrimidines can bind to a duplex DNA containing natural bases either in a parallel or antiparallel fashion (Fig. 2)^13,36–41^. Usually, purines in the TFO are believed to bind to purines in the duplex DNA (G-GC or A-AT) by adopting antiparallel conformation^37^. Similarly, pyrimidines in the TFO are believed to bind to purines in the duplex DNA (C-GC and T-AT) by adopting parallel conformations^36^. However, G in the third strand was observed to bind to G in the duplex strand (G-GC) by adopting both parallel^13,36,37^ and antiparallel^39^ conformations (Fig. 2). Similarly, A and C^+^ (protonated cytosine) were found to adopt the antiparallel conformation to produce the A-AT and C^+^-GC triplexes respectively^13^ (Fig. 2). In another X-ray study^40^, TFOs containing C^+^ and 5-bromouracil were found to bind to the purine-rich (G and A) duplex DNA by adopting only parallel conformation. Notably when a nucleotide in the TFO binds to a duplex DNA containing purine-rich or pyrimidine-rich nucleotides by making Hoogsteen interactions, it produces a parallel triplet and when it binds by making reverse Hoogsteen interactions, it produces an antiparallel triplet (Fig. 2). In the antiparallel triple, the TFO undergoes a 180° rotation with respect to the nucleotide with which it is making reverse Hoogsteen interaction^13,36–41^. However, the generation of triple helical DNA by involving artificial nucleotides has not yet been explored. Therefore, it is necessary to understand the structural and energetic details of triplets formed by involving the above eight second-generation artificial nucleobases (Fig. 1).

For these reasons, the bindings of natural purines (G and A) and pyrimidines (C, T) to the complementary three hydrogen-bonded artificial base pairs, such as PZ, JV, BS, and XK were studied by using a reliable dispersion-corrected density functional theoretic (DFT) method. Subsequently, the bindings of artificial purines (P, J, B, and X), and pyrimidines (Z, V, S, and K) to the three hydrogen-bonded Watson–Crick GC pair were studied by the same method. We have not considered the binding of artificial nucleobases with the AT pair, as such triplets will be less stable compared to those formed by involving the GC pair. In doing so, the natural and synthetic nucleobase containing TFOs were allowed to bind to each base (purine and pyrimidine) in a base pair. For example, P was binding to both G and C in the GC pair to produce P-GC and GC-P triplets respectively. Further, to identify the most stable triplets, the bindings of the triplex-forming nucleobases with different base pairs were studied by considering their parallel and antiparallel conformations (Fig. 2). The most stable triplets were further solvated in 5 explicit water molecules and the effect of consecutive stacking of triplets were also evaluated. It is thus expected that this study will help to realize the potential of the second-generation artificial nucleotides in forming triple helical DNA for various DNA technological applications.

## Computational methodology

The geometries of P, Z, J, V, B, S, X, K, G, C, A, and T were optimized by using the ωb97XD dispersion corrected density functional theoretic method^42–45^ and 6 − 31 + G* basis set^46^ in the aqueous medium. In these molecules, the glycosidic bonds (dR, Fig. 1) were replaced by H atoms. Subsequently, the geometries of all triplets were optimized by the same level of theory in the aqueous medium. The integral equation formalism of the polarized continuum model (IEFPCM)^47,48^ was used to model the aqueous medium. The vibrational analysis was undertaken to ensure that all the optimized structures had real vibrational frequencies.

To obtain more accurate energies, the ωb97XD/AUG-cc-pVDZ level of theory was used for the single-point energy calculations in the aqueous medium. This method was found to produce energies, which are equivalent to the energies obtained by the highly accurate and computationally expensive CBS-Q method^25^. The zero-point energies obtained at the ωb97XD/6–31 + G* level of theory were considered to be valid for the ωb97XD/AUG-cc-pVDZ level of theory. All calculations were carried out by using the Gaussian-09 (G09) program^49^. The structures were visualized by using the GaussView-05 program^50^.

The binding energies $\left({E}_{BE}\right)$ of different triplets were calculated by using the Eq. (1).

1 $$E_{BE} = E_{A - BC} - \left( {E_{A} + E_{B} + E_{C} } \right)$$

here, ${E}_{ABC}$ refers to the zero-point energy (ZPE)- corrected total energy of the A-BC triplex. Similarly, ${E}_{A}$, ${E}_{B}, {and E}_{C}$ refer to the ZPE-corrected total energies of A, B, and C monomers.

To consider the effect of microsolvation on base pair triplets, the geometries of the most stable triplets were further optimized in the presence of 5 explicit water molecules. The implicit solvent model (IEFPCM) was kept intact during these geometry optimizations. The binding energies of microsolvated complexes were calculated by using the Eq. (1). To consider the effect of consecutive base pair stacking, the geometry of the C-JV/C-JV complex (/ refers to stacking interaction) was optimized in the implicit aqueous medium. As the C-JV triplet is the most stable one, the C-JV/C-JV complex was considered here to understand the impact of sequence specificity on the structure and energy of the C-JV/C-JV complex. The results were compared with those of C+-GC/T-AT complex. The stacking interaction energy (E_SE_) was calculated by using the Eq. (2).

$$E_{SE} = E_{A - BC/D - EF} - \left( {E_{A - BC} + E_{D - EF} } \right)$$

where ${E}_{A-BC/D-EF}$ is the ZPE-corrected total energy of the A-BC/D-EF complex, ${E}_{A-BC}$ is the ZPE-corrected total energy of the A-BC triplet, and ${E}_{D-EF}$ is the ZPE-corrected total energy of the D-EF triplet. As the ωb97XD/AUG-cc-pVDZ level of theory produces accurate energies, we will discuss ZPE-corrected binding energies obtained by this method only.

## Results and discussions

### M-PZ and PZ-M triplets (M = G, C, A, and T)

**Figure 3:**
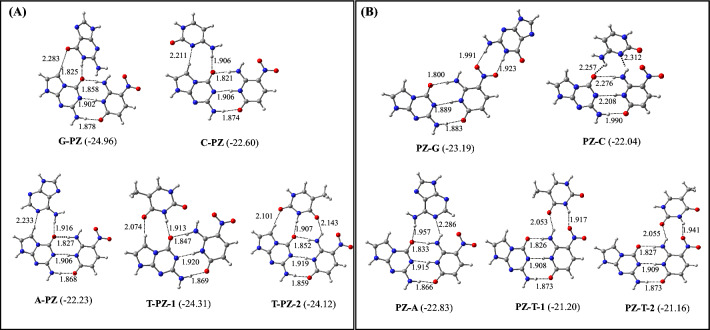
The optimized structures of M-PZ and PZ-M triplets (M = G, C, A, and T). The binding energies (kcal/mol) of different triplets obtained at the wB97X-D/AUG-cc-pVDZ level of theory are provided in parentheses. Two isoenergetic T-PZ complexes are shown as T-PZ-1 and T-PZ-2. Similarly, two isoenergetic PZ-T complexes are shown as PZ-T-1 and PZ-T-2.

During geometry optimizations, it was found that triplex forming bases (M) adopt different conformations to produce M-PZ and PZ-M triplets (M = G, C, A, and T). The optimized structures of the most stable M-PZ and PZ-M triplets are shown in Fig. 3. Their binding energies are presented in Table 1. The optimized structures of all such triplets are illustrated in Figures S1 and S2 (Supplementary Information). From Fig. 3A, it is evident that G binds with P to form the G-PZ triplet by adopting antiparallel conformation, while C and A bind with P to form C-PZ and A-PZ triplets respectively by adopting parallel conformations. These triplets are stabilized by two hydrogen bonds. Interestingly, the binding of T with P by adopting both parallel and antiparallel conformations formed isoenergetic T-PZ triplets (Fig. 3A). Hence T may adopt both parallel and antiparallel conformations to form T-PZ triplets (Fig. 3A). If we compare the binding energy of these triplets, it follows the order G-PZ > T-PZ > C-PZ > A-PZ (Fig. 3A, Table 1). This suggests that the binding of G with P in the duplex DNA containing the PZ pair would produce the most stable triplex, which would be about 1–3 kcal/mol more stable than other triplets of this series (Fig. 3A, Table 1). Although these triplets are stabilized primarily by direct hydrogen bonding interactions, it is likely that secondary interactions, such as dipole–dipole, and long-range electrostatic interactions are contributing favorably to the stability of these triplets^26^.

**Table 1:** The zero-point energy-corrected binding energies of M-PZ and PZ-M (M = G, C, A, and T) triplets.

Triplets	Binding energies (kcal/mol)	
	ωB97XD/6 − 31 + G*	ωB97XD/Aug-cc-pVDZ
G-PZ	− 23.57	− 24.96
C-PZ	− 21.34	− 22.60
A-PZ	− 20.80	− 22.23
T-PZ-1	− 23.04	− 24.31
T-PZ-2	− 22.17	− 24.12
PZ-G	− 22.12	− 23.19
PZ-C	− 21.26	− 22.04
PZ-A	− 21.73	− 22.83
PZ-T-1	− 20.36	− 21.20
PZ-T-2	− 20.31	− 21.16

It is found that G binds to Z in the PZ pair to form the PZ-G triplet by adopting antiparallel conformation. However, C and A adopt parallel conformations to bind with Z to form PZ-C and PZ-A triplets respectively (Fig. 3B). Interestingly, T can adopt both parallel and antiparallel conformations to produce PZ-T triplets in similar manure as obtained for the T-PZ triplets (Fig. 3B). However, in the PZ-T triplets, T binds to Z by adopting a twisted conformation resulting non-planar complexes (Figs. 3 and S3). This happened due to the electrostatic repulsion between the O atom of the NO_2_ group of Z and the O2 or O4 atom of T. If we compare the binding energy of all PZ-M triplets, it follows the order PZ-G > PZ-A > PZ-C > PZ-T (Fig. 3B, Table 1). This indicates that the binding of G with Z in the duplex containing the PZ pair will produce the most stable triplet, which is about 1–3 kcal/mol more stable than other triplets of this series. Further, if we compare G-PZ and PZ-G triplets, the former is found to be about 1 kcal/mol more stable than the latter. Therefore, G will prefer to bind to P rather than Z in the PZ pair. In an earlier study, the binding energy of the PZ base pair was computed to be − 16.45 kcal/mol by using the ωB97XD/AUG-cc-pVDZ level of theory in the aqueous medium^25,26^. As the binding energy of the G-PZ triplet is about − 25.0 kcal/mol, it appears that the binding of G to the PZ pair would enhance the stability of the PZ pair by about 9 kcal/mol. It is also evident that the highest stability of the G-PZ triplet is arising because of the strong primary hydrogen bonding interactions and weak secondary interactions, such as dipole–dipole, and long-range electrostatic interactions^26^.

In an earlier X-ray study^40^, it was proposed that the extended purine-rich sequences on a duplex DNA strand would lead to the formation of a triple helix, where the third strand will run parallel to the purine strand. However, no such trend is found here. For example, if a duplex DNA contains artificial purine-rich (e.g. P) sequences, the third strand containing natural purines (G and A) would bind to the duplex strand by adopting both parallel and antiparallel conformations (Fig. 3A). Similarly, the third strand containing natural pyrimidines (C and T) would also bind to the artificial purine-rich duplex strand by adopting both parallel and antiparallel conformations (Fig. 3A). It was proposed that the conversion of duplex to triplex would lead to partial unwinding of DNA due to mainly lower twist and higher base pair opening angles^40,41^. It was shown that the binding of C^+^ to G in the GC pair would elongate the hydrogen bonds between G and C^40^. However, in all triplets containing PZ pairs, (except the PZ-C), the PZ base pair hydrogen bonds lie between 1.8–1.9 Å, which is the same as those found for the isolated PZ pair^26^. Slightly increased hydrogen bonds between P and Z obtained in the PZ-C triplet are mainly due to the electrostatic repulsion between the carbonyl group of C and NO_2_ group of Z. Therefore, M-PZ and PZ-M triplets would not lead to base-pair opening in DNA in agreement with an earlier X-ray study involving the G-GC triplet^38^.

### M-JV and JV-M triplets (M = G, C, A, and T)

**Figure 4:**
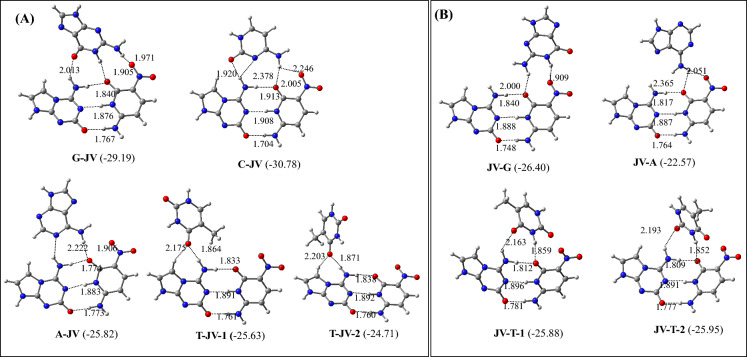
Optimized structures of the M-JV and JV-M triplets (M = G, C, A, and T). The binding energies (kcal/mol) obtained at the ωB97XD/AUG-cc-pVDZ level of theory are shown in parentheses.

The optimized structures of the most stable M-JV and JV-M (M = G, C, A, and T) triplets are shown in Fig. 4. The binding energies of these triplets are presented in Table 2. All optimized structures of these triplexes are shown in Figures S4 and S5. From Fig. 4A, it is evident that G binds to J in the JV base pair by adopting antiparallel conformation. In the G-JV triplet, G makes three hydrogen bonds with both J and V. However, C and A bind with J in the JV pair by adopting parallel conformations. In the C-JV and A-JV triplets, C and A make three and two hydrogen bonds with both J and V respectively. Similar results were obtained for the G-GC triplet by using an X-ray study^38^. It was found that G can bind with both G and C^38^, and the resulting complex would be more stable compared to the binding of G with only G in the GC pair^50^.

**Table 2:** The zero-point energy-corrected binding energies of M-JV and JV-M (M = G, C, A, and T) triplets.

Triplets	Binding energies (kcal/mol)	
	ωB97XD/6–31 + G*	ωB97XD/Aug-cc-pVDZ
G-JV	− 27.94	− 29.19
A-JV	− 24.13	− 25.82
C-JV	− 29.35	− 30.78
T-JV-1	− 24.17	− 25.63
T-JV-2	− 23.22	− 24.71
JV-G	− 27.89	− 29.05
JV-A	− 21.34	− 22.57
JV-T-1	− 24.94	− 25.88
JV-T-2	− 24.91	− 25.95

Although T can bind to J in the JV pair by adopting both parallel and antiparallel conformations, the former conformation of T makes a slightly more stable (by about 0.9 kcal/mol) T-JV triplet (Fig. 4A). However, both the T-JV triplets are non-planar due to the twisted conformation of T (Figs. 4 and S3). As the Hoogsteen face of J contains only one hydrogen bond donor group, it can make at most one hydrogen bond with T by engaging either its O2 or O4 atom. For this reason, T gets twisted to facilitate the NH_2_(J)-O2(T) or NH_2_(J)-O4(T) hydrogen bond. However, the twisted T-JV triplet is unlikely to be formed in the DNA. If we compare the binding energy of the above triplets, it follows the order, C-JV > G-JV > A-JV ≥ T-JV (Table 2, Fig. 4A). Hence, C will bind to J to form the most stable C-JV triplet. The higher stability of the C-JV triplet is arising because of the strong primary hydrogen bonding interactions and weak secondary interactions, such as dipole–dipole, and long-range electrostatic interactions^26^. Interestingly, the binding mode of C-JV triplet is similar to the experimental binding mode of G-GC triplet^38^.

Among the JV-M triplets (M = G, C, A, and T), G, A, and T bind to V by making two hydrogen bonds each (Fig. 4B). In the JV-G and JV-A triplets, G and A adopt antiparallel conformations. Interestingly, all possible structures of the JV-C triplet, where C binds with V converted to C-JV triplet (Fig. 4A). This implies that C will bind to both J and V in the C-JV triplet. However, due to the isoenergetic nature of the JV-T-1 and JV-T2 complexes, T can adopt both parallel and antiparallel conformations to bind with V (Fig. 4B). To avoid the electrostatic repulsion between the O2 or O4 atom of T with the O atom of NO_2_ group of V, T acquires a twisted conformation in the JV-T1 and JV-T2 triplets (Figs. 4, S3). The binding energy of these complexes follows the order JV-G > JV-T > JV-A (Fig. 4B, Table 2). This implies that G can bind more tightly with V compared to other triplex-forming natural bases. Further, if we compare the binding energy of M-JV and JV-M (M = G, C, A, and T) triplets, it is evident that C would prefer to bind to J to produce the most stable C-JV triplet (Table 2, Fig. 4). In an earlier study^25^, the binding energy of the JV base pair by employing the ωB97XD/AUG-cc-pVDZ level of theory in the aqueous medium was computed to be − 18.61 kcal/mol. As the binding energy of the C-JV triplet is about − 31 kcal/mol (Table 2), it can be assumed that the binding of C to J in the JV pair would enhance the stability of the JV pair by about 12 kcal/mol. Further, as in all the above triplets, the hydrogen bond lengths between J and V lie between 1.7–1.9 Å, which are the same as obtained for isolated JV base pair^25^, these triplets will not lead to the base pair opening.

### M-BS and BS-M triplets (M = G, C, A, and T)

**Figure 5:**
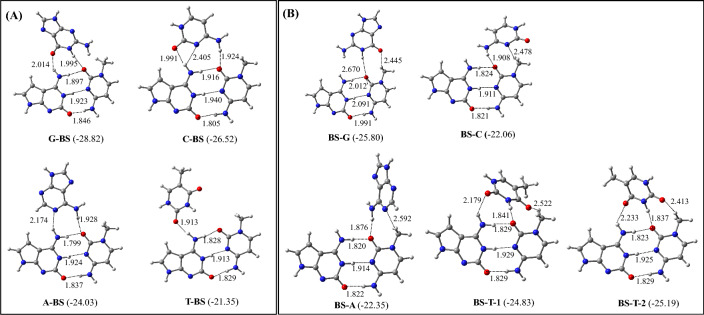
Structures of the most stable (**A**) M-BS and (**B**) BS-M (M = G, C, A, and T) triplets. The binding energies (kcal/mol) obtained at the ωB97XD/AUG-cc-pVDZ level of theory are shown in parentheses.

The most stable M-BS and BS-M (M = G, C, A, and T) triplets are depicted in Fig. 5. Their binding energies are presented in Table 3. The optimized structures of all triplets involving BS pair are presented in Figures S6 and S7. It is found that G binds to B in the BS pair by adopting antiparallel conformation, whereas C, A, and T bind with B by adopting parallel conformation (Fig. 5A). The G-BS triplet is stabilized by two hydrogen bonds in the reverse Hoogsteen pattern, where G interacts with both B and S. Similarly, in the C-BS triplet, C interacts with both B and S by making three hydrogen bonds, while A and T interact only with B in the A-BS and T-BS triplets by making two and one hydrogen bond respectively. Interestingly, among all M-BS triplets, T-BS is nonplanar because of the slightly twisted structure of T (Figs. 5 and S3). As the G-BS triplet is the most stable one, which is about 2–7 kcal/mol more stable than other triplets of this series (Fig. 5A, Table 3), it is likely that the G-BS triplet will only be formed in the triple helical DNA. In addition to the primary hydrogen bonds, the G-BS triplet is also stabilized by secondary dipole–dipole interactions and long-range electrostatic interactions^26^.

**Table 3:** The zero-point energy-corrected binding energies of M-BS and BS-M (M = G, C, A, and T) triplets.

Triplets	Binding energies (kcal/mol)	
	ωB97XD/6–31 + G*	ωB97XD/Aug-cc-pVDZ
G-BS	− 24.24	− 28.82
C-BS	− 24.81	− 26.52
A-BS	− 22.31	− 24.03
T-BS	− 19.94	− 21.35
BS-G	− 24.23	− 25.80
BS-C	− 20.66	− 22.06
BS-A	− 20.89	− 22.35
BS-T-1	− 23.48	− 24.83
BS-T-2	− 23.69	− 25.19

In the BS-G triplet, G interacts with S by adopting antiparallel conformation. However, C and A interact with S by adopting parallel conformation (Fig. 5B). However, T can adopt both parallel and antiparallel conformations to produce BS-T-1 and BS-T-2 triplets respectively due to their identical binding energies (Fig. 5B, Table 2). Interestingly, in these triplets, while G, C, and A interact only with S, T makes hydrogen bonds with both S and B (Fig. 5B). As A and T adopt highly twisted conformations (Figs. 5 and S3) to make favorable hydrogen bonding interactions with S, these triplets are unlikely to be formed in the triple helical DNA. However, as the BS-G triplet is the most stable one, which is about 1–3 kcal/mol more stable than other triplets of this series (Table 3), its formation would be abundant in the triple helical DNA. If we compare the G-BS and BS-G triplets, it is clear that the former is more stable than the latter by about 3 kcal/mol. Hence, G will prefer to bind to B rather than S to yield the G-BS triplet. In an earlier study, the binding energy of the BS base pair was computed to be − 17.15 kcal/mol by using the ωB97XD/AUG-cc-pVDZ level of theory in the aqueous medium^25^. As the binding energy of the G-BS triplet is about − 29 kcal/mol, it appears that the binding of G to B in the BS pair would enhance the stability of the BS pair by about 12 kcal/mol. Further, as in all the above triplets, the hydrogen bond lengths between B and S lie between 1.8–1.9 Å, which are the same as obtained for isolated BS base pair^25^, the formations of these triplets will not lead to the base pair opening.

### M-XK and XK-M triplets (M = G, C, A, and T)

**Figure 6:**
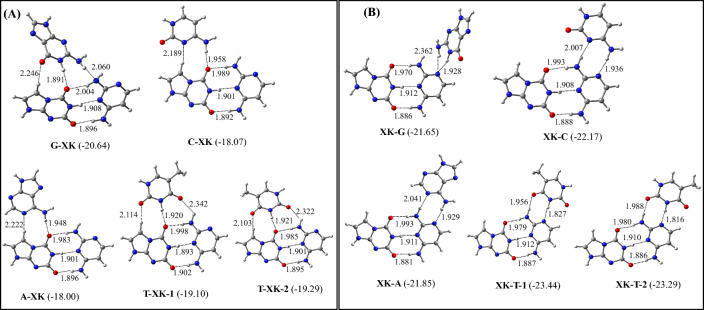
Structures of the most stable (**A**) M-XK and (**B**) XK-M (M = G, C, A, and T) triplets. The binding energies (kcal/mol) obtained at the ωB97XD/AUG-cc-pVDZ level of theories are shown in parentheses.

The optimized structures of the most stable M-XK and XK-M triplets are shown in Fig. 6. The binding energies of these triplets are presented in Table 4. The optimized structures of all these triplets are shown in Figures S8 and S9. It is found that G binds to X in the antiparallel conformation, while C, and A bind to X in the parallel conformation. However, T can bind to X by adopting both parallel and antiparallel conformations (Fig. 6A). In the G-XK and T-XK triplets, G and T are found to make hydrogen bonds with both X and K, while C and A interacted only with X. Interestingly, T adopts a slightly twisted conformation to make favorable hydrogen bonding interactions with X to form non-planar T-XK triplets (Figs. 6 and S3). As in the normal B-DNA, a propeller twist of − 11.4 deg. is acceptable^51^, the T-XK-1 and T-XK-2 triplets are likely to be formed in the triple helical DNA. Among the M-XK triplets, the G-XK triplet is found to be the most stable one, which is about 1–2 kcal/mol more stable compared to other triplets of this series (Fig. 6A, Table 4). Hence, the occurrence of G-XK in the triple helical DNA would be higher compared to other triplets of this series.

**Table 4:** The zero-point energy-corrected binding energies of M-XK and XK-M (M = G, C, A, and T) triplets.

Triplets	Binding energies (kcal/mol)	
	ωB97XD/6–31 + G*	ωB97XD/Aug-cc-pVDZ
G-XK	− 19.59	− 20.64
C-XK	− 16.59	− 18.07
A-XK	− 16.42	− 18.00
T-XK-1	− 17.67	− 19.10
T-XK-1	− 17.79	− 19.29
XK-G	− 20.12	− 21.65
XK-C	− 20.27	− 22.17
XK-A	− 19.77	− 21.85
XK-T-1	− 21.25	− 23.44
XK-T-2	− 21.08	− 23.29

The G, C, and T are found to bind with K by making two hydrogen bonds each to form the XK-G, XK-C, and XK-A triplets respectively. In these triplets, they have acquired antiparallel conformation (Fig. 6B). Due to the identical energies of the XK-T-1 and XK-T-2 triplets, T can bind to K by adopting both parallel and antiparallel conformations. Among the XK-M triplets, XK-T is found to be the most stable, which is about 2–8 kcal/mol more stable than other triplets of this series (Table 4, Fig. 6B). The XK-T triplet is also about 3 kcal/mol more stable than the G-XK triplet (Table 4, Fig. 6). This indicates that T would prefer to bind with K instead of X to generate the XK-T triplet. Although the X-KT triplet is slightly non-planar (propeller twist <  − 11.4 deg.)^51^ (Figs. 6 and S3), it is likely to be formed in the triple helical DNA. In an earlier study, the binding energy of the XK base pair by employing the ωB97XD/AUG-cc-pVDZ level of theory in the aqueous medium was computed to be − 11.72 kcal/mol ^25^. As the binding energy of the XK-T triplet is about − 23.44 kcal/mol, it appears that the binding of T to the XK pair would enhance the stability of the XK pair by about 11 kcal/mol. Further, as in all the above triplets, the hydrogen bond lengths between X and K lie between 1.9–2.0 Å, which are the same as obtained for isolated XK base pair ^25^, the formations of these triplets will also not lead to the base pair opening.

If we compare all triplets involving M-PZ, PZ-M, M-JV, JV-M, M-BS, BS-M, M-XK, and XK-M, (Figs. 3, 4, 5, 6), it is evident that the binding of C with J in the JV pair would yield the most stable C-JV triplet. As in the C-JV triplet, C (pyrimidine) interacts with both J (purine) and V (pyrimidine), it can be proposed that the hydrogen bonding interactions between pyrimidine-purine and pyrimidine-pyrimidine would play an important role in stabilizing a semisynthetic triple helical DNA.

### N-GC and GC-N triplets (N = P, J, B, and X)

**Figure 7:**
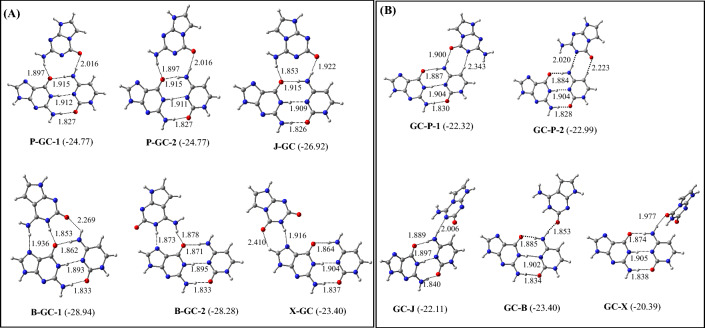
Structures of the most stable (**A**) N-GC and (**B**) GC-N (M = P, J, B, and X) triplets. The binding energies (kcal/mol) obtained at the ωB97XD/AUG-cc-pVDZ level of theories are shown in parentheses.

The optimized structures of the most stable N-GC and GC-N (N = P, J, B, and X) triplets are illustrated in Fig. 7. The binding energies of these triplets are presented in Table 5. All the possible optimized structures of these triplets are shown in Figures S10 and S11. It is found that P can bind to G by making both parallel and antiparallel conformations to produce the P-GC-1 and P-GC-2 triplets respectively. Interestingly, these two triplets are isoenergetic (Table 5). Although J binds to G by making antiparallel conformation to produce the J-GC triplet, B binds to G by making both parallel and antiparallel conformations (Fig. 7A). The B-GC-1 triplet where B adopts antiparallel conformation is slightly more stable (by 0.66 kcal/mol) than the B-GC-2 in which B binds to G by making parallel conformation. Further, in the P-GC and J-GC triplets, P and J make one hydrogen bond each with both G and C (Fig. 7A), while in the B-GC triplet, B makes two hydrogen bonds with G and one hydrogen bond with C. The binding energy of these triplets follows the order B-GC > J-GC > P-GC > X-GC (Fig. 7, Table 5). Hence, the binding of B to G in the GC base pair will produce the most stable triplet, which would be about 2–6 kcal/mol more stable than the binding of other artificial purines to G (Fig. 7A, Table 5).

**Table 5:** The zero-point energy-corrected binding energies of N-GC and GC-N (N = P, J, B, X) triplets. Zero-point energy-corrected O-GC and GC-O (O = Z, V, S, and K) triplets are also provided.

Triplets	Binding energies (kcal/mol)	
	ωB97XD/6–31 + G*	ωB97XD/Aug-cc-pVDZ
P-GC-1	− 23.29	− 24.77
P-GC-2	− 23.29	− 24.77
J-GC	− 25.36	− 26.92
B-GC-1	− 27.24	− 28.94
B-GC-2	− 26.26	− 28.28
X-GC	− 21.97	− 23.40
GC-P-1	− 20.93	− 22.32
GC-P-2	− 20.75	− 22.99
GC-J	− 20.75	− 22.11
GC-B	− 21.97	− 23.40
GC-X	− 18.95	− 20.39
Z-GC-1	− 25.53	− 30.24
Z-GC-2	− 28.59	− 30.22
V-GC-1	− 28.61	− 30.25
V-GC-2	− 27.96	− 29.70
S-GC	− 24.12	− 25.69
K-GC-1	− 19.58	− 20.82
K-GC-2	− 19.07	− 20.44
GC-Z	− 19.09	− 20.49
GC-V	− 20.42	− 21.87
GC-S	− 21.34	− 20.44
GC-K1	− 20.52	− 21.65
GC-K1	− 20.44	− 21.63

P also binds to C to produce the GC-P triplet by adopting both parallel and antiparallel conformations (Fig. 7B). The antiparallel conformation of P makes a slightly more (0.67 kcal/mol) stable GC-P triplet compared to the parallel conformation of P (Fig. 7B). However, J, B, and X bind to C by adopting parallel conformation. In all these triplets, artificial purines interacted only with C by making two or one hydrogen bond (Fig. 7B). However, GC-J, GC-B, and GC-X are non-planar due to the twisted conformation of J, B, and X respectively. As mentioned earlier, these artificial purines adopt twisted conformations to produce favorable hydrogen bonding interactions with the GC pair. If we compare the binding energy of these triplets, it follows the order GC-B > GC-P > GC-J > GC-X (Table 5, Fig. 7B). This indicates that B will bind strongly with C to produce the GC-B triplet. If we compare the B-GC and GC-B triplets, it is evident that the former is about 5.5 kcal/mol more stable than the latter. Hence, B would prefer to bind with G instead of C to produce the most stable B-GC triplet (Fig. 7, Table 5). Further, as in all the above triplets, the hydrogen bond lengths between G and C lie between 1.8–1.9 Å, which are the same as obtained for isolated GC base pair ^25^, the formations of these triplets will not lead to the base pair opening.

### O-GC and GC-O triplets (O = Z, V, S, and K)

**Figure 8:**
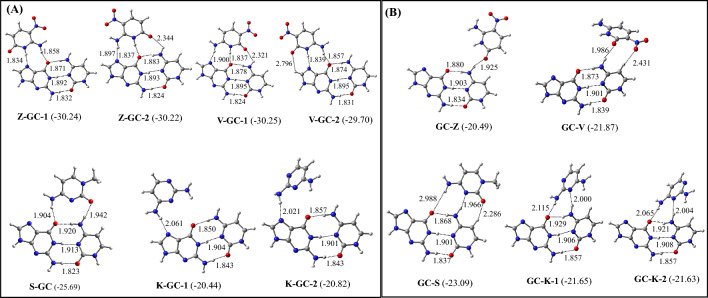
Structures of the most stable (**A**) O-GC and (**B**) GC-O (O = Z, V, S, and K) triplets. The binding energies (kcal/mol) obtained at the ωB97XD/AUG-cc-pVDZ level of theories are shown in parentheses.

The optimized structures of the most stable O-GC and GC-O triplets (O = Z, V, S, and X) are shown in Fig. 8. The binding energies of these triplets are provided in Table 5. All possible structures of these triplets are shown in Figures S12 and S13. It is found that the binding of Z to G in the parallel and antiparallel conformations would produce isoenergetic Z-GC-1 and Z-GC-2 triplets respectively. (Fig. 8, Table 5). In the parallel conformation, Z makes two strong Hoogsteen hydrogen bonds with G, while in the antiparallel conformation, it makes three reverse Hoogsteen hydrogen bonds with G. Similarly, V can bind to G to produce the V-GC triplet by adopting both parallel and antiparallel conformations. The parallel conformation of V binds to both G and C by making three hydrogen bonds (V-GC-1), while its antiparallel conformation binds to G only by making three hydrogen bonds (V-GC-2) (Fig. 8A). However, the parallel conformation of V can produce a slightly (by 0.55 kcal/mol) more stable V-GC triplet compared to its antiparallel conformation. Similarly, in the S-GC triplet, S binds to both G and C by adopting a parallel conformation, whereas K can adopt both parallel and antiparallel conformations to produce the K-GC triplets (Fig. 8A). However, the antiparallel conformation of K (K-GC-2) produces a slightly more stable (by 0.38 kcal/mol) K-GC triplet. Despite of its higher stability, the formation of K-GC-2 in the triple helical DNA is unlikely due to a higher propeller twist.

If we compare the binding energy of these triplets, it follows the order V-GC ≅ Z-GC > S-GC > K-GC (Table 5, Fig. 8A). Hence, the binding of Z and V to G in the GC pair would produce the most stable triplets in this series. Like other triplets, the Z-GC and V-GC triplets are also stabilized by hydrogen bonding, long-range electrostatic, and dipole–dipole interactions.

Among the GC-O triplets, Z binds to C in the parallel conformation, while V and S bind to C in the antiparallel conformation to produce the GC-Z, GC-V, and GC-S triplets respectively (Fig. 8B). However, K can adopt both parallel and antiparallel conformations to bind with C to produce the GC-K-1 and GC-K-2 triplets respectively (Fig. 8B). However, as GC-K triplets (GC-K-1 and GC-K-2) are non-planar with a higher propeller twist (>-11.4 deg.)^51^ (Figs. 8B and S3) their occurrences in the triple helical DNA would be unlikely. If we compare the binding energy of these triplets, it follows the order GC-S > GC-V ≥ GC-K > GC-Z (Fig. 8B, Table 5). Hence, S will preferably bind to C to produce the GC-S triplet. If we compare O-GC and GC-O triplets, it is evident that the binding of V (pyrimidine) and Z (pyrimidine) with both G (purine) and C (pyrimidine) in the GC pair would be highly favored to produce the pyrimidine-purine- and pyrimidine-pyrimidine-like triplets.

Interestingly, among all the eight second-generation artificial nucleobases binding to the GC pair (Figs. 7 and 8, Table 5), the Z-GC and V-GC triplets are the most stable. Therefore, it is quite likely that artificial pyrimidines (Z and V) containing TFOs will form the most stable complex by binding to G in the GC pair containing DNA duplex. Further, as the binding energy of the GC pair is about − 15.80 kcal/mol^25^, the binding of Z or V to the GC pair would enhance the stability of the GC pair by about 14 kcal/mol.

### Effects of explicit water molecules and stacking interaction on base pair triplets

To understand the effects of explicit water molecules on the stability of the most stable base pair triplets, interactions of 5 water molecules with the C-JV, Z-GC, and V-GC triplets were undertaken by using the same levels of theory. The 5 water molecules constitute the first solvation shell around these triplets and make hydrogen bonds with all hydrogen bond donors and acceptors (except with N9H, which will be replaced by a glycosidic bond in the triple helical DNA)^52^. Similarly, to understand the effect of base pair stacking on the C-JV triplet, consecutive C-JV triplets were stacked. It should be mentioned that three or more consecutive stacked triplets were structurally characterized earlier^53^. Recently, 11 consecutive U-AU base triplets containing RNA were observed by using the X-ray study (PDB ID 6SVS)^53^. In this study, a C^+^-GC triplet was also observed^54^. To compare the binding energy of the above semisynthetic triplets (C-JV, Z-GC, and V-GC) with that of the X-ray structures of U-AU and C^+^-GC RNA triplets, the coordinates of the latter complexes were extracted from the protein data bank (PDB ID 6SVS) and subjected to geometry minimizations. Subsequently, the U-AT and C^+^-GC RNA triplets were converted to T-AT and C-GC DNA triplets respectively. The binding energies of these semisynthetic and natural triplets (RNA and DNA) are presented in Table 6. It is found that the C-JV triplet is about 10 kcal/mol more stable than the U-AU, T-AT, and C-GC triplets (Table 6). However, it is about 3 kcal/mol less stable than the C^+^-GC triplet. The higher stability of the C^+^-GC triplet is presumably due to the H3 proton of cytosine. Similar energetic trends are also obtained for the Z-GC and V-GC triplets (Table 6). Interestingly, the solvation of C-JV and V-GC triplets in five explicit water molecules enhanced their stability by about 21 kcal/mol, while the stability of solvated Z-VC is increased by 16 kcal/mol (Table 6 and Figure S14). This implies that the microsolvation of semisynthetic triplets would enhance their stability appreciably. It also indicates that J-VC and V-GC would be isoenergetic in explicit water molecules.

**Table 6:** Binding energies of different triplets obtained at different levels of theory.

Triplets	Binding energy (kcal/mol)		Stacking interactions (kcal/mol)	
	ωB97XD/6–31 + G*	ωB97XD/AUG-cc-pVDZ	ωB97XD/6–31 + G*	ωB97XD/AUG-cc-pVDZ
C-JV	− 29.35	− 30.78	–	–
Z-GC	− 28.59	− 30.22	–	–
V-GC	− 28.61	− 30.25	–	–
U-AU	− 18.83	− 20.59	–	–
T-AT	− 18.90	− 20.62	–	–
C-GC	− 19.46	− 20.72	–	–
C^+^-GC	− 31.57	− 33.61	–	–
C-JV-5H_2_O	− 53.06	− 51.94	–	–
Z-GC-5H_2_O	− 47.17	− 46.01	–	–
V-GC-5H_2_O	− 53.16	− 51.81	–	–
C-JV/C-JV	− 77.02	− 79.67	− 18.35	− 18.16
C^+^-GC/T-AT	− 73.64	− 78.11	− 23.17	− 23.88

**Figure 9:**
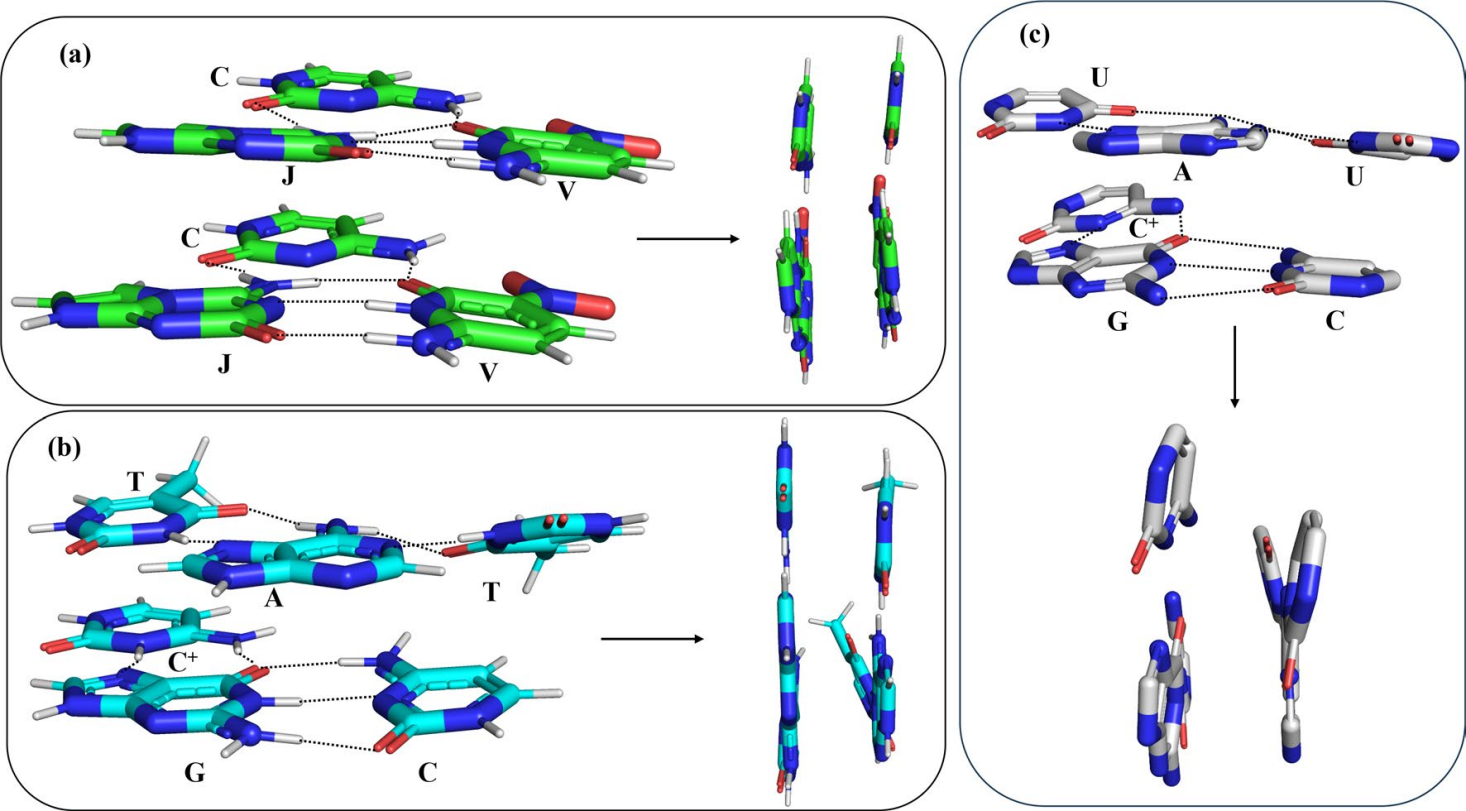
The optimized structures of the (**a**) C-JV/C-JV and (**b**) C^+^-GC/T-AT complexes. (**c**) The X-ray structure of the C^+^-GC/U-AU complex is also shown for structural comparison. The left and right panels in (**a**) and (**b**) represent the top and side views of the C-JV/C-JV and C^+^-GC/T-AT complexes respectively. The top and side views of the C^+^-GC/U-AU complex are also shown in (**c**).

It is further found that the binding energy of the C-JV/C-JV complex is about -80 kcal/mol, which is about 2 kcal/mol more stable than the C^+^-GC/T-AT complex (Table 6). This stability mainly arises due to the strong hydrogen bonding interactions between C, J, and V. Interestingly, the stacking interaction of the C^+^G-C/T-AT complex is found to be more negative than that of the C-JV/C-JV complex. This is because; the CH_4_ group of T protrudes toward the GC pair, thereby making a slightly more favourable van der Waals interaction (Fig. 9). Interestingly, it is noticed that the planar base pair triplets, such as C-JV, C^+^-GC, and T-AT became non-planar in the C-JV/C-JV and C^+^-GC/T-AT stacked structures to facilitate optimum hydrogen bonding and stacking interactions (Fig. 9a,b). In the X-ray structure of the C^+^-GC/U-AU complex, the C^+^-GC and U-AU triplets were also observed to be non-planar (Fig. 9c) (PDB ID 6SVS) due to a slightly more propeller twist^51^. A rise of about 3.4 Å is obtained for both C-JV/C-JV and C^+^-GC/T-AT complexes, which corresponds to that observed for B-DNA^48^. However, an average rise of 2.9 Å was obtained for the triple helical RNA containing 11 U-AU triplets^54^. These results indicate that semisynthetic triple helical DNA can be generated by using artificial nucleotides. It is also possible that other artificial bases that are not considered here but can make hydrogen bonding interactions with their complimentary counterparts^55–58^ may also form semisynthetic triple helical DNA. Therefore, a detailed study on triple helical DNA by considering a full-length DNA containing artificial nucleotides may yield more interesting results.

## Conclusions

It is found that the binding of natural bases with artificial base pairs would produce stable triplets whose binding energies lie between ~ − 21.0 to ~ − 31.0 kcal/mol. Among these triplets, the C-JV triplet is found to be the most stable one. It is further found that the microsolvation of the C-JV triplet in 5 explicit water molecules would enhance its stability by 21 kcal/mol. Therefore, it is likely that the natural nucleobase (mainly C) containing TFOs can bind to the semisynthetic duplex DNA containing JV pair to form a stable triple helical DNA. It is also revealed that the artificial bases can bind with the natural GC base pair to produce stable triplets whose binding energies lie between ~ − 20.0 to ~ − 30.0 kcal/mol. Among these triplets, the Z-GC or V-GC triplet is found to be the most stable, each of which possesses a binding energy of about -30.0 kcal/mol. The microsolvation of these triplets in 5 explicit water molecules further enhanced their stability by ~ 16–21 kcal/mol. Consecutive stacking interactions between these triplets would even further enhance their stability. Therefore, it is likely that the TFOs containing artificial nucleobases (mainly Z and V) can bind to the duplex DNA containing GC pair to form stable semisynthetic triple helical DNA. It is also revealed that in the semisynthetic triple helical DNA, the triplex-forming nucleobases may adopt both parallel and antiparallel conformations. However, the consideration of a full-length triple helical DNA containing one or more artificial bases or base pairs would yield more interesting results.

### Supplementary Information

Supplementary Information.

## Data Availability

All data are presented in the manuscript and Supporting Information.
