# Supplementary material for: Structure and stability of different triplets involving artificial nucleobases: clues for the formation of semisynthetic triple helical DNA
Source: Sci Rep. 2023 Nov 7;13:19246. doi: 10.1038/s41598-023-46572-4 (PMC10630353; doi:10.1038/s41598-023-46572-4)
Supplement: Supplementary file 1 — Supplementary Information. [file 41598_2023_46572_MOESM1_ESM.docx]

Supplementary Material

**Structures and Stabilities of Different Triplets Involving Artificial Nucleobases: Clues for the Formation of Semisynthetic Triple Helical DNA**

**N.R. Jena^a*^ and P.K. Shukla^b^**

^a^Discipline of Natural Sciences, Indian Institute of Information Technology, Design, and Manufacturing, Dumna Airport Road, Khamaria, Jabalpur-482005, India

^a^Department of Physics, Assam University, Silchar-788 011, Assam, India

*Corresponding Author’s E-mail: [nrjena@iiitdmj.ac.in](mailto:nrjena@iiitdmj.ac.in)

Fig. S1: The optimized Structures of M-PZ (M=G, C, A, and T) triplets. The ZPE-corrected binding energies (kcal/mol) obtained at the ωB97XD/6-31+G* (ωB97XD/AUG-cc-pVDZ) level of theory are also shown.

Fig. S2: The optimized Structures of PZ-M (M=G, C, A, and T) triplets. The ZPE-corrected binding energies (kcal/mol) obtained at the ωB97XD/6-31+G* (ωB97XD/AUG-cc-pVDZ) level of theory are also shown.

Fig. S3: The side views of different non-planar triplets.

Fig. S4: The optimized Structures of M-JV (M=G, C, A, and T) triplets. The ZPE-corrected binding energies (kcal/mol) obtained at the ωB97XD/6-31+G* (ωB97XD/AUG-cc-pVDZ) level of theory are also shown.

Fig. S5: The optimized Structures of JV-M (M=G, C, A, and T) triplets. The ZPE-corrected binding energies (kcal/mol) obtained at the ωB97XD/6-31+G* (ωB97XD/AUG-cc-pVDZ) level of theory are also shown.

Fig. S6: The optimized Structures of M-BS (M=G, C, A, and T) triplets. The ZPE-corrected binding energies (kcal/mol) obtained at the ωB97XD/6-31+G* (ωB97XD/AUG-cc-pVDZ) level of theory are also shown.

Fig. S7: The optimized Structures of BS-M (M=G, C, A, and T) triplets. The ZPE-corrected binding energies (kcal/mol) obtained at the ωB97XD/6-31+G* (ωB97XD/AUG-cc-pVDZ) level of theory are also shown.

Fig. S8: The optimized Structures of M-XK (M=G, C, A, and T) triplets. The ZPE-corrected binding energies (kcal/mol) obtained at the ωB97XD/6-31+G* (ωB97XD/AUG-cc-pVDZ) level of theory are also shown.

Fig. S9: The optimized Structures of XK-M (M=G, C, A, and T) triplets. The ZPE-corrected binding energies (kcal/mol) obtained at the ωB97XD/6-31+G* (ωB97XD/AUG-cc-pVDZ) level of theory are also shown.

Fig. S10: The optimized Structures of N-GC (N=P, J, B, and X) triplets. The ZPE-corrected binding energies (kcal/mol) obtained at the ωB97XD/6-31+G* (ωB97XD/AUG-cc-pVDZ) level of theory are also shown.

Fig. S11: The optimized Structures of GC-N (N=P, J, B, and X) triplets. The ZPE-corrected binding energies (kcal/mol) obtained at the ωB97XD/6-31+G* (ωB97XD/AUG-cc-pVDZ) level of theory are also shown.

Fig. S12: The optimized Structures of O-GC (O=Z, V, S, and K) triplets. The ZPE-corrected binding energies (kcal/mol) obtained at the ωB97XD/6-31+G* (ωB97XD/AUG-cc-pVDZ) level of theory are also shown.

Fig. S13: The optimized Structures of GC-O (O=Z, V, S, and K) triplets. The ZPE-corrected binding energies (kcal/mol) obtained at the ωB97XD/6-31+G* (ωB97XD/AUG-cc-pVDZ) level of theory are also shown.

Fig. S14: Optimized structures of different (a) base pair triplets and (b) microsolvated triplets. Hydrogen bonds are indicated by dotted lines.
